# Association of familial macular degeneration with specific genetic markers: a case report

**DOI:** 10.1186/s13256-015-0765-7

**Published:** 2015-11-24

**Authors:** Yoshinori Takayanagi, Masami Ashida, Mayumi Go, Mai Gunji, Izuru Sato, Shigeaki Kato, Masato Miyashita

**Affiliations:** 1 CARNAMED Eye Clinic, Sapporo S1 Building 3F, Nishi4-20-5, Minami1-jo, Chuouku, Sapporo, Hokkaido 060-0807 Japan; 2 DAL-DNA Analysis Laboratory, Co. Ltd, Sapporo North, Building 3F, Nishi2-8-1, Kita7-jo, Kitaku, Sapporo, Hokkaido 060-0807 Japan; 3 Research Center, Jyoban Hospital, 57 Kaminodai, Jyoban, Kamiyunagayamachi, Iwaki, Fukushima 972-8322 Japan

**Keywords:** Age-related macular degeneration, *ARMS2* gene, Susceptibility gene locus

## Abstract

**Introduction:**

Age-related macular degeneration is a serious visual disorder of the central retina and was recently reported to be associated with genetic background. Here we describe a genetic link to early onset age-related macular degeneration in members of an Asian family.

**Case presentation:**

A 73-year-old Asian woman developed age-related macular degeneration in the fifth decade of her life and her 49-year-old daughter developed age-related macular degeneration. Because of the family history and the early onset, family members were tested for two single nucleotide polymorphism variants (rs10490924 and rs11200638) at a recently identified susceptibility locus for age-related macular degeneration. Both alleles in the 73-year-old woman were of the high-risk variants (T/T for rs10490924 and A/A for rs11200638), and her two daughters and a grandson each carried the risk variants (T and A) one on each allele.

**Conclusions:**

In a case where multiple family members had early onset age-related macular degeneration, we found two high-risk single nucleotide polymorphism variants in the age-related macular degeneration susceptibility locus, suggesting the combination of the known single nucleotide polymorphism variants as a potent age-related macular degeneration diagnostic indicator.

## Introduction

Age-related macular degeneration (AMD) is a serious visual disorder of the central retina and it is prevalent in developed countries [1, 2]. In Asian patients, exudative (wet) AMD occurs more frequently than geographic atrophy (dry) AMD seen in European patients with late AMD [3, 4]. Choroidal vasculopathy around the macula triggers abnormal neovascularization, leading to serious hemorrhaging and exudation [5]. For most European patients, without such hemorrhages, retinal thinning is accompanied by atrophy of the retinal pigment epithelium [5].

Irrespective of similarities in phenotypic defects among AMD types, the mechanisms underlying the development of AMD are not well understood. However, from the familial incidence of AMD, genetic background has been suspected and, accordingly, genes associated with AMD have been identified for both the dry and wet types of AMD [6–8]. Recent genetic association studies have been successful in identifying several AMD risk-associated single nucleotide polymorphism (SNP) variants, and the SNP variants are specific to AMD type, reflecting different genetic backgrounds among races. Through genome-wide association studies (GWASs) on large populations of patients with AMD that included independent groups, several SNP susceptibility variants were identified in genes encoding complement factor H (*CFH*), high-temperature requirement factor A1 (*HTRA1*) and age-related maculopathy susceptibility 2 (*ARMS2*), as well as in the regions for TNFSF10A-LOC389641 and REST-C4orf14-POLR2B-IGFBP7 for dry-type [9] and for wet-type AMD [10, 11].

Diagnosis of AMD is certified for patients over 50 years of age, but there is also a group of patients with AMD with early onset [1, 2]. For such patients, hereditary SNP variation in loci associated with AMD is assumed. We report here an Asian case of familial AMD with early onset bearing both of the known high-risk SNP variants (rs10490924 and rs11200638 in *ARMS2* and *HTRA1*, respectively) [10, 11] in patients with AMD, suggesting a genetic association between the two SNPs in AMD onset.

## Case presentation

**Fig. 1:**
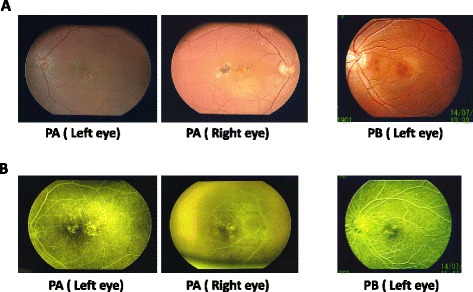
Color fundus photographs and fluorescein angiograms. **a** Fundus photographs of the patients (PA and PB are the family members depicted in Fig. 2). The images for PA were taken in 2007 and those for PB in 2014. **b** Fluorescein angiograms for PA taken in 1996 and PB in 2014. Choroidal neovascularization, hyperpigmentation and reticular pseudodrusen were seen as representative markers for classical wet type of age-related macular degeneration. *PA* Patient A, *PB* Patient B

**Fig. 2:**
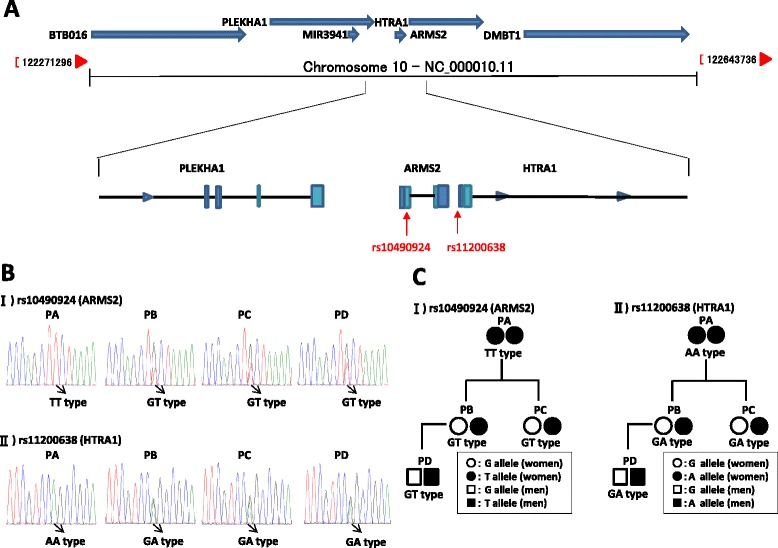
Single nucleotide polymorphism variant analysis of a family with age-related macular degeneration in the age-related macular degeneration susceptibility locus around the *ARMS2* and *HTRA1* genes. **a** Two loci for single nucleotide polymorphism variants (rs10490924 and rs11200638) in the *ARMS2* and *HTRA1* genes on chromosome 10q26. **b** Sequence electropherograms of the rs10490924 and rs11200638 loci for each of the family members. **c** Pedigree of the family with genetic variants in rs10490924 and rs11200638 loci on chromosome 10q26. *PA* Patient A, *PB* Patient B, *PC* Patient C, *PD* Patient D

A 73-year-old Asian woman consulted 19 years ago (1996) requesting examination of her right eye, since both eyes bore pterygium and her left eye was diagnosed as having wet-type AMD in another ophthalmological clinic 23 years ago (1992); she is designated as Patient A (PA) in the rest of this case report. She was reported to have blurriness in her central visual field in the early part of the third decade of her life. Intraocular pressure was 14 mmHg in her right eye and 12 mmHg in her left eye, and no overt abnormalities were seen in the cornea or the crystalline lens in either eye by slit lamp test in 1996. A fluorescein angiogram (FAG) in 1996 and a fundus examination in 2007 of her right eye showed classical wet-type AMD with choroidal neovascularization, hyperpigmentation and reticular pseudodrusen (Fig. 1). Her best-corrected visual acuity was 20/25 for her right eye and 20/30 for her left eye in 1992, but these acuities gradually decreased over several years to 20/300 for her left eye and, in 2003, they reached less than 20/400 in both eyes. A SNP survey was conducted last year for the recently reported susceptibility loci *ARMS2* and *HTRA1* for wet AMD (Fig. 2a). Both SNP variants in this patient (PA) were high-risk alleles: T/T for rs10490924 in the coding region (a mutation that converts alanine 69 into serine in the ARMS2 protein; A69S) and A/A for rs11200638 in the promoter of the *HTRA1* gene (Fig. 2a, b) [10, 11]. After these findings from SNP analysis, one of PA’s daughters, who was 49 years of age (Patient B; PB), visited our hospital for a SNP survey; she also manifested blurred central vision in her left eye and was afraid of AMD. Her best-corrected visual acuity was 20/20 for her right eye and 20/25 for her left eye. Intraocular pressure was 16 mmHg for her right eye and 15 mmHg for her left eye; neither eye exhibited overt abnormalities by slit lamp examination. However, in a fundus examination, abnormality of the retinal pigment epithelium was detected, as well as reticular pseudodrusen, and she was diagnosed as having classic wet AMD in 2014 (Fig. 1). To prevent AMD progression, an anti-vascular endothelial growth factor (VEGF) antibody, Eylea (aflibercept ophthalmic), for the left eye of PB was administered in 2014. After 10 months, she reported that blurring was ameliorated and the best-corrected visual acuity for her left eye was consistently found to be 20/20. PB and her younger sister, at 45 years of age (Patient C; PC), took the SNP survey (Fig. 2b, c). High-risk variants were found in both high-risk loci: T for rs10490924 and A for rs11200638 [11, 12]. The younger sister (PC) and the grandson (Patient D; PD) had no manifestations of any AMD-related visual defect.

## Discussion

AMD is a common eye condition among patients aged 50 years and older [1, 2]. The onset and progression of AMD are diverse, but AMD, per se, is generally not serious enough to cause complete blindness [1, 2]. However, the loss of central vision from damage to the macula threatens the visual abilities that support every aspect of normal life, since the center of the field of view is distorted [1–4].

Although common features are detected among patients with AMD, the molecular basis of AMD onset and development remains to be uncovered. Moreover, effective treatments and drugs to prevent AMD are still in clinical trials [12, 13]. In this respect, investigation of genetic variants in patients with AMD in large populations is a promising strategy for identifying molecular targets for intervention in AMD, as it is well established that familial history is a strong predictor of AMD. Among the AMD susceptibility loci [6–11], we identified two SNP variants that mutated A69 of the ARMS2 protein into alanine (A69S) (T/T for rs10490924) and a variation with high risk (A/A) in the promoter of the *HTRA1* gene at rs11200638, since these SNP variants are predictive of AMD incidence with high odds ratios (2.86) by GWAS analyses of large numbers of patients with AMD [10]. Moreover, a mechanistic link between the ARMS2 A69S mutation and AMD progression has been illustrated [10]. Both of the tested high-risk alleles (T for rs10490924 and A for rs11200638) were found in all of the Asian family members, and all of them had had dry eye since adolescence. As the relationship among the multiple susceptibility gene loci delineated by several independent groups remains to be studied in terms of development and onset of AMD, our findings are intriguing because they suggest mutual associations among the identified susceptibility gene loci. Further assessment of other SNP variants related to AMD may add more genetic information. This study has many limitations because it is a case report and needs more examination to assess our conclusions. It is also evident that other factors underlie the onset and development of AMD. However, the present case study represents a rational approach to provide a prevention strategy for those at risk for AMD, particularly for those with a familial AMD history.

## Conclusions

An Asian family with early onset AMD bore the known high-risk SNP variants in both *ARMS2* and *HTRA1*; the presence of both SNPs could be an indicator for early onset AMD.

## Consent

Written informed consent was obtained from the patients for publication of this case report and any accompanying images. Copies of written consents are available for review by the Editor-in-Chief of this journal.

## Abbreviations

AMD: Age-related macular degeneration
*ARMS2*: Age-related maculopathy susceptibility 2
GWAS: Genome-wide association study
*HTRA1*: High-temperature requirement factor A1
PA: Patient A
PB: Patient B
PC: Patient C
SNP: Single nucleotide polymorphism
